# Do shorter inter‐stimulus intervals in the go/no‐go task enable better assessment of response inhibition?

**DOI:** 10.1111/sjop.12679

**Published:** 2020-10-04

**Authors:** Akira Hasegawa, Noboru Matsumoto, Yuko Yamashita, Keisuke Tanaka, Jun Kawaguchi, Tetsuya Yamamoto

**Affiliations:** ^1^ Faculty of Human Relations Tokai Gakuin University 5‐68 Naka‐kirino Kakamigahara City, Gifu 504‐8511 Japan; ^2^ Division of Psychology Faculty of Arts Shinshu University Asahi 3‐1‐1 Matsumoto, Nagano 390‐8621 Japan; ^3^ Graduate School of Integrated Arts and Sciences Tokushima University 1‐1, Minamijosanjima‐cho Tokushima 770‐8502 Japan; ^4^ Graduate School of Education Joetsu University of Education 1‐Yamayashiki‐machi, Joetsu‐shi Niigata 943‐8512 Japan; ^5^ Department of Psychology Otemon Gakuin University 2‐1‐15 Nishiai, Ibaraki City, Osaka 567‐8502 Japan; ^6^ Graduate School of Informatics Nagoya University Furo‐cho, Chikusa‐ku Nagoya Aichi 464‐8601 Japan; ^7^ Graduate School of Technology, Industrial and Social Sciences Tokushima University 1‐1, Minamijosanjima‐cho Tokushima 770‐8502 Japan

**Keywords:** Executive function, go/no‐go, impulsive action, impulsivity, inhibition, Stroop

## Abstract

Young, Sutherland, and McCoy indicated that a Go/No‐Go Task (GNG) becomes more difficult as the inter‐stimulus intervals (ISIs) becomes shorter. However, is the number of commission errors under extremely short ISIs a useful metric for assessing response inhibition? This study challenges the assumption that a shorter ISI in the GNG enables better assessment of response inhibition. University students (N = 213) completed the GNG, the Conners Continuous Performance Test 3rd Edition (CCPT), and the Modified Stroop Task. The GNG comprised four blocks of 400, 600, 800, and 1000 ms ISIs, whereas the stimulus presentation was fixed at 250 ms. Consistent with Young *et al*., shorter ISIs in the GNG resulted in more commission errors. In the block with the shortest ISI, participants also failed more frequently in responses in go trials than in the other blocks, which appears to increase in error variance of commission errors. Consistent with this interpretation, the association between the number of commission errors in the block with 400 ms ISI and CCPT performance was weaker than those between the number of commission errors in other blocks and CCPT performance. It is concluded that using the number of commission errors in the condition with extremely short ISIs in the GNG might be inappropriate for assessing response inhibition.

## Introduction

A Go/No‐Go Task (GNG) is a simple task in which participants are instructed to respond to one class of stimtuli (go trials) but to withhold responses to another class of stimuli (no‐go trials). Commission errors in GNG tasks that indicate erroneous responses in the no‐go trials have frequently been used as indices of inhibition, and especially response inhibition, in studies of the domain of the executive function (Snyder, Miyake & Hankin, 2015; Tiego, Testa, Bellgrove, Pantelis & Whittle, 2018; Wright, Lipszyc, Dupuis, Thayapararajah & Schachar, 2014). Additionally, commission errors have also been used as indices of behavioral impulsivity, particularly impulsive actions, in studies of the domain of impulsivity (Grant & Chamberlain, 2014; MacKillop, Weafer, Gray, Oshri, Palmer & de Wit, 2016; Weafer & de Wit, 2014). Response inhibition represents the ability to suppress a prepotent motor response (Tiego *et al*., 2018), while impulsive action refers to the inability to inhibit this response (Grant & Chamberlain, 2014).

Previous studies have suggested that the number of commission errors in the GNG serves as a useful assessment tool in psychopathology. Studies using the number of commission errors in the GNG and in its variants, such as the Conners Continuous Performance Test (CCPT) and the Sustained Attention to Response Task as indices showed that response inhibition was impaired among individuals with Autism Spectrum Disorder, Attention‐Deficit/Hyperactivity Disorder, Schizophrenia, Bipolar Disorder, Major Depressive Disorder, Obsessive‐Compulsive Disorder, Substance‐Related and Addictive Disorders, and Personality Disorders (Wright *et al*., 2014). These findings indicated that the number of commission errors in the GNG could reflect common deficits of the executive function across various psychiatric disorders, although it remains somewhat uncertain why response inhibition is related to these disorders. Furthermore, previous twin studies suggested that the number of commission errors in the GNG was moderately heritable (Bezdjian, Tuvblad, Wang, Raine & Baker, 2014; Kuntsi, Rogers, Swinard *et al*., 2006), indicating that it is plausible that impaired response inhibition may be a genetic risk factor or a genetic deficit associated with several psychiatric disorders.

Typically, the GNG has been conducted using non‐emotional stimuli, such as colored circles and letters of the alphabet, while sometimes this task is conducted using emotional stimuli, including positive and negative adjectives and facial expressions. This article focuses on only non‐emotional GNG because performance in the executive function tasks using an emotional stimulus can reflect not only impairments in executive function but also altered emotional processing (Snyder *et al*., 2015).

It is also essential to consider how the GNG was implemented in previous studies varied from study to study in terms of a number of factors, such as stimulus type, go/no‐go ratio, the time during which stimuli were presented, and inter‐stimulus interval (ISI). This variability seems to be related to the difficulty of the task because, in previous studies that used the GNG with several characteristics yet retained a sample with the same attribute, the number of commission errors varied among studies. For example, among previous studies of university students, Enge, Sach, Reif, Lesch, Miller and Fleischhauer (2020) reported about 30% of commission error rate in the GNG, while Caswell, Bond, Duka and Morgan (2015) and Hasegawa, Somatori, Nishimura, Hattori and Kunisato (2019) showed about 3% of commission error rate. Hasegawa *et al*. (2019) also noted that about half of the participants in their study did not make any commission errors. This finding indicated that their GNG settings, in which green and red circles were used as go and no‐go stimulus, respectively, go/no‐go ratio was 7:3, stimulus presentation was fixed at 500, and ISI was at 1000 ms, was too easy for university students.

If most participants make few or no errors in the no‐go trials, the number of commission errors is an insensitive measure of individual differences in response inhibition (i.e., floor effect). The difficulty of the GNG is crucial in the case of adult samples in particular because previous studies have shown that during the growth period from childhood to young adulthood, commission errors decrease with increasing age (Bezdjian *et al*., 2014; Eigsti *et al*., 2006). Researchers should examine suitable settings in the GNG to assess individual differences of response inhibition in a study with an adult sample.

One of the critical variables related to the number of commission errors in the GNG is ISI. It is assumed that the shorter the ISI is, the more commission errors the participants make. Young, Sutherland and McCoy (2018, study 1) conducted a study with undergraduate students to examine the effects of differences in ISI as well as go/no‐go ratio on the number of commission errors. These researchers set the stimulus presentation duration of go and no‐go stimulus to 250 ms, and then manipulated the ISI from 200 ms to 1000 ms per participant. Results showed a linear relationship between shorter ISI and increased commission errors, although this relationship was somewhat moderated by the go/no‐go ratio. The authors suggested that, with respect to manipulations of the ISI, the shortest ISIs (i.e., 200 ms in their study) produced the highest number of commission errors in the GNG.

However, it is not known whether increased commission errors in the GNG with extremely short ISI enable better assessment of response inhibition. Under an extremely short ISI condition, it is plausible that some participants simply have insufficient time to identify whether the stimulus is a go or no‐go stimulus before it disappears. In this case, participants may simply press a key in time with the presentation of any stimulus without identifying the type of stimulus; others may be confused by the situation in which the stimulus changes quickly and cannot make any response in some consecutive trials (i.e., freezing). Either case would prevent researchers from assessing response inhibition accurately. Previous studies have not examined these *deleterious* effects of extremely short ISI in the GNG on the appropriateness of the number of commission errors for measuring response inhibition.

Therefore, the present study investigated the appropriate ISI for the GNG for measuring response inhibition. This study uses the GNG composed of four blocks in which the durations of presented go and no‐go stimulus were fixed, but the ISIs differed from block to block. The appropriateness of ISIs is evaluated with respect to the distribution of the number of commission and omission errors (i.e., errors in the go trials), and the relationships between the number of commission errors and other inhibition measures. In terms of the commission errors, we consider that a block with a desirable ISI will produce more commission errors and result in a normal distribution of the number of commission errors than other blocks with different ISIs, and fewer participants will make no commission errors in that block than in other blocks. In terms of the omission errors, we propose that in a block with desirable ISI, the number of omission errors should be relatively low, and fewer participants should produce one or more omission errors than in other blocks because some proportion of omission errors should reflect participants’ confusion, as described above. Furthermore, the number of commission errors in a block containing a desirable ISI in the GNG should show stronger positive associations with the number of commission errors in the CCPT, a measure of response inhibition that has been shown to have high reliability and validity (Conners, 2014) and also with the number of incongruent‐trial errors in the modified Stroop task (MST), a standard measure of inhibition than in the other blocks.

In the GNG, white and red circles were used as go and no‐go stimuli, respectively. Each stimulus was presented for 250 ms. The ISIs were set to 400, 600, 800, and 1000 ms in each block, respectively, and the order of the blocks was randomized among participants. Although Young *et al*. (2018, study 1) showed that 200 ms ISI produced the highest number of commission errors in terms of manipulating ISI, our preliminary experiment suggested that it is too difficult for university students to respond after identifying whether the stimulus is a go or no‐go stimulus in the block with only 200 ms ISI. Therefore, this study set the shortest ISI to 400 ms.

## Methods

### Participants

Undergraduate and graduate students aged from 18 to 30 were recruited at Joetsu University of Education, Nagoya University, Tokai Gakuin University, and Tokushima University in Japan. Two‐hundred and thirteen students (Mean age 19.8 years, *SD* = 1.4; age ranged from 18 to 26 years) participated in this study. We recruited students of the age range described above to avoid any age effects. All participants were Japanese, with the exception of one participant who was Vietnamese.

### Measures

#### Go/No‐Go Task (GNG)

The GNG based on the procedure adopted by Gutiérrez‐Cobo, Cabello and Fernández‐Berrocal (2017) and Hasegawa *et al*. (2019) was used. This task was composed of go trials, in which a white circle with a diameter of about 5 cm appeared in the center of a black screen, and no‐go trials, in which a red circle of the same size appeared. The participants were instructed to press the “b” key as quickly as possible in the go trials and not to press any key in the no‐go trials. The GNG began with the presentation of a fixation cross for 1000 ms, followed by a blank screen for 2000 ms. Then, the go and the no‐go trials were presented in random order.

Participants completed a practice phase composed of 10 trials with an equal number of go and no‐go trials before completing the test trial phase. During practice trials, the stimulus appeared for 500 ms, with 1000 ms between trials, during which time the participants could respond. After confirming that participants understood the task, participants completed the test trial phase, which was composed of 4 blocks of 120 trials. During each trial in all blocks, the stimulus appeared for 250 ms. However, ISIs varied across blocks. ISIs in each block were 400, 600, 800, and 1000 ms, respectively, and the order of the blocks was randomized among participants. Seventy percent of trials were go trials, and 30% were no‐go trials. We calculated the number of times each participant made erroneous responses in no‐go trials (i.e., commission errors) and the number of times they could not respond in go trials (i.e., omission errors) in each block. In addition, although the issue is not directly related to our research question, some readers may be interested in the impact of ISI on reaction times (RTs). Therefore, mean RTs in the correct go trials were calculated for each block.

#### Conners Continuous Performance Test 3rd Edition (CCPT)

The CCPT is a reliable and valid test developed by Conners (2014). In this task, 16 uppercase letters of the alphabet are displayed in the center of a white screen for 250 ms. The ISI changes to 1, 2, or 4 seconds for every 20 trials. Participants were instructed to press the space bar as quickly as possible when a letter other than an “X” was displayed (go trials), but to inhibit response when an “X” was shown (no‐go trials). The test phase composed of 361 trials was administered after a brief practice phase, such that 80% of the test trials were go trials, and the others were no‐go trials. The first trial was not scored, and the number of the commission errors in the remainder of the trials was used to assess response inhibition.

There are specific differences between the GNG and the CCPT such as the type of stimuli (i.e., colored circles vs. letters of the alphabet), the number of go stimuli used (i.e., only one stimulus vs. 15 stimuli), and the fluctuation of the ISIs (i.e., no fluctuation in identical blocks vs. three kinds of ISIs in successive trials). Increased number of go stimuli and the fluctuation of the ISIs in the CCPT may tax more cognitive resources than the GNG. For this reason, the increased variance in the number of commission errors in the CCPT might reflect other constructs than response inhibition compared to the GNG. However, the CCPT had good validity and reliability (Conners, 2014), and therefore it is a sufficient indicator of construct validity.

#### Modified Stroop Task (MST)

The task based on the procedure adopted by Altamirano, Miyake and Whitmer (2010) was used. Three Kanji characters (a kind of Japanese written character) that were blue, yellow, and red, respectively, were used. The size of these characters was 70 points, and each character was also presented in a blue, yellow, or red color. Characters were displayed one by one in each trial in the center of a black screen. Participants were instructed to press the “1” key as quickly as possible when the color of the character on the screen was blue, or “2” when the color was yellow, or “3” when the color was red while disregarding the meaning of the character displayed. The character on the screen disappeared when participants responded, and after ISIs of 500 ms, the next character appeared. This task comprised 75% congruent trials in which the color and meaning of characters which appeared on the screen matched, and 25% incongruent trials in which they did not match. The MST began with the presentation of a fixation cross for 1000 ms, followed by a blank screen that was presented for 2000 ms. Next, the congruent and incongruent trials were presented in random order. After a practice phase composed of 24 trials, participants completed four blocks of 48 trials. The number of erroneous responses was calculated for incongruent trials in the test phase. The number of incongruent‐trial errors was used to assess inhibition.

### Procedure

Students who were interested in this study were individually invited to the authors’ laboratory. First, we obtained their informed consent for participating in the study. Then, participants completed the GNG, the MST, and the CCPT in a fixed order. The three tasks were administered on a computer screen using 1366 × 768 pixels. The GNG and the MST were administered via Inquisit 5 (Millisecond Software, LLC), and the CCPT was administered a special software for conducting this task (Multi‐Health Systems Inc.). Participants could take a short break between tasks and between blocks in the GNG and the MST. After completing the three tasks, the participants responded to several questionnaires that were unrelated to the purpose of this study. After the completion of the study, participants were debriefed and were given a gift certificate worth 1500 yen (approximately US$13). The study took approximately 1 hour for each participant to complete. The Ethics Committee of Tokai Gakuin University approved this study.

### Statistical analysis

A multilevel Poisson regression analysis was conducted using Mplus 8.3 (Muthén & Muthén, 1998–2019). *Z*‐tests were used to compare correlations using R. The violin plot was depicted using the BellCurve for Excel (SSRI Corporation). All other analyses were conducted using SPSS ver. 23 (IBM Corporation). Missing data were handled by the pairwise method. Zero‐order Pearson's correlations were computed between normally distributed variables. The Z‐test for comparing two overlapping correlations was conducted based on dependent groups, according to Hittner, May and Silver (2003). Because the number of commission errors and omission errors in the GNG was count variables, we conducted a multilevel Poisson regression analysis with maximum likelihood estimation with robust standard error (MLR) for predicting these variables. One‐way Friedman test and pairwise Wilcoxon signed‐rank test with Bonferroni correction were conducted to examine the significant between block differences in RTs since parts of these variables were highly skewed.^1^

## Results

The CCPT data of four participants, who fell asleep during the CCPT, were excluded. In addition, the MST data of four participants were excluded: three participants gave answers indicating the content of characters but not their colors, and one participant fell asleep during the task.

**Fig 1:**
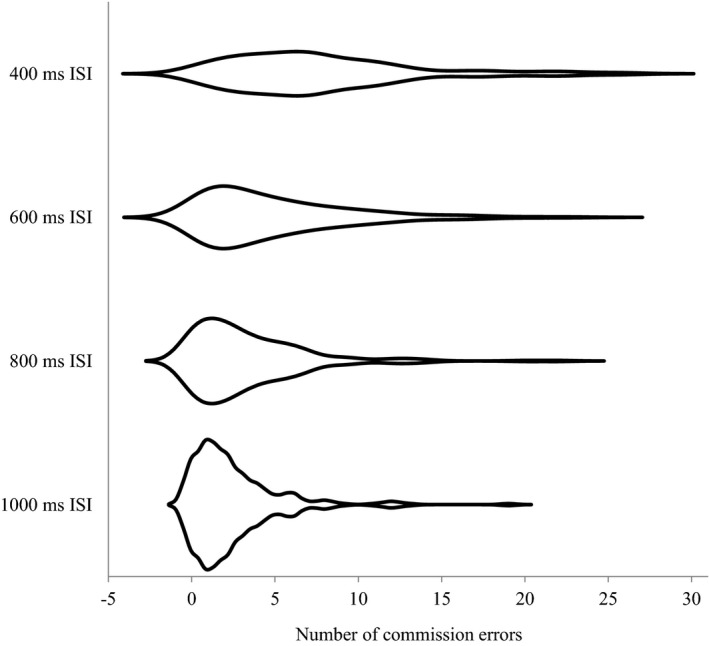
Distribution of the number of commission errors in each block with different ISIs of the GNG (all *ns* = 213).

Descriptive statistics for each measure are displayed in Table 1. Among the number of commission errors in the GNG, those in the blocks with 800 ms and 1000 ms ISIs were highly skewed. As shown in Fig. 1, participants who made no or few commission errors increased as the ISIs in blocks became longer, resulting in a skewed distribution of the number of commission errors in blocks with longer ISIs. The number of participants who made no commission errors was 8 (4%) in the block with 400 ms ISI, 18 (8%) in that with 600 ms ISI, 38 (18%) in that with 800 ms ISI, and 40 (19%) in that with 1000 ms ISI, respectively.

**Table 1:** Descriptive statistics for the measures used in this study

	Mean	Median (IQR)	Range	Skewness	Kurtosis
GNG with 400 ms ISI					
Commission errors	7.60	7.00 (4.00–10.00)	0.00 – 26.00	1.19	1.44
Omission errors	2.07	1.00 (0.00–3.00)	0.00 – 28.00	3.63	18.63
Reaction time	305.80	304.15 (285.90–323.51)	190.65 – 418.81	0.36	1.05
GNG with 600 ms ISI					
Commission errors	4.80	3.00 (1.00–7.00)	0.00 – 23.00	1.43	2.14
Omission errors	0.52	0.00 (0.00–0.00)	0.00 – 18.00	7.23	59.84
Reaction time	307.91	302.29 (288.26–327.60)	195.77 – 429.17	0.51	1.54
GNG with 800 ms ISI					
Commission errors	3.30	2.00 (1.00–5.00)	0.00 – 22.00	2.09	6.52
Omission errors	0.15	0.00 (0.00–0.00)	0.00 – 5.00	5.27	36.24
Reaction time	311.45	308.85 (293.02–327.74)	211.17 – 458.24	1.13	4.37
GNG with 1000 ms ISI					
Commission errors	2.45	2.00 (1.00–3.00)	0.00 – 19.00	2.44	8.75
Omission errors	0.12	0.00 (0.00–0.00)	0.00 – 6.00	6.89	54.71
Reaction time	317.35	311.86 (296.39–334.68)	246.17 – 507.12	1.77	7.43
CCPT					
Commission errors	17.38	17.00 (11.00–22.00)	0.00 – 48.00	0.70	0.31
MST					
Incongruent‐trial errors	5.32	4.00 (2.00–7.00)	0.00 – 25.00	1.49	2.91

Note

GNG = Go/No‐Go Task; CCPT = Conners Continuous Performance Test 3rd Edition; MST = Modified Stroop task; ISI = Inter‐stimulus interval; *ns* = 213, 209, and 209 for GNG data, CCPT data, and MST data, respectively.

A multilevel Poisson regression analysis with MLR was conducted to examine the between block differences in the number of commission errors. Conditions (i.e., three dummy variables: blocks with 400 ms ISI (0) vs. 600 ms ISI (1), those with 400 ms ISI (0) vs. 800 ms ISI (1), and those with 400 ms ISI (0) vs. 1000 ms ISI (1)) were used as fixed factors and the number of commission errors as the dependent variable. Results showed that participants made significantly more commission errors in the block with 400 ms ISI than in the other blocks (Table 2). In addition, the magnitude of each parameter estimated in Table 2 suggested that participants made more commission errors as the ISI in the block became shorter.

**Table 2:** The results of multilevel Poisson regressions with the blocks as independent variables and the number of commission errors and omission errors in the GNG as dependent variables (both ns = 213)

Parameter	Estimate	*SE*	*Z*	*p*
Dependent variable: commission errors				
Intercept	2.03	0.05	41.17	<.001
Block [400 ms ISI (0) vs. 600 ms ISI (1)]	−0.46	0.05	−9.52	<.001
Block [400 ms ISI (0) vs. 800 ms ISI (1)]	−0.84	0.06	−14.15	<.001
Block [400 ms ISI (0) vs. 1000 ms ISI (1)]	−1.13	0.06	−17.62	<.001
Dependent variable: omission errors				
Intercept	0.73	0.12	6.30	<.001
Block [400 ms ISI (0) vs. 600 ms ISI (1)]	−1.39	0.23	−6.17	<.001
Block [400 ms ISI (0) vs. 800 ms ISI (1)]	−2.62	0.21	−12.32	<.001
Block [400 ms ISI (0) vs. 1000 ms ISI (1)]	−2.83	0.34	−8.24	<.001

Note

GNG = Go/No‐Go Task; ISI = Inter‐stimulus interval.

A similar multilevel Poisson regression analysis was conducted to examine the between block differences in the number of omission errors. Results showed that participants made significantly more omission errors in the 400 ms ISI block than in other blocks (Table 2). Furthermore, as shown in Table 2, the magnitude of each parameter estimate indicated that participants tended to make more omission errors in the 600 ms ISI block than in the blocks with 800 and 1000 ms ISIs, whereas they made a similar number of omission errors in the last two blocks.

The number of participants that made one or more omission errors was 122 (57%) in the block with 400 ms ISI, 43 (20%) in that with 600 ms ISI, 22 (10%) in that with 800 ms ISI, and 14 (7%) in that with 1000 ms ISI, respectively. These distributions indicate that many participants made one or more errors in the block with the 400 ms ISI while most participants did not make any errors in the other blocks. This resulted in a significant effect of blocks on the number of omission errors found in the multilevel Poisson regression analysis.

Next, we examined between block differences in the strength of association between the number of commission errors in the GNG and performances in the other two inhibition tasks. A multilevel Poisson regression analysis with MLR was conducted for using conditions in the GNG, the number of mean centered commission errors in the CCPT and interactions between conditions and CCPT performance as fixed factors, and the number of commission errors in the GNG as the dependent variable. Table 3 shows the results of this analysis. The main effects of conditions in the GNG and CCPT performance, as well as all interactions between each GNG condition and CCPT performance, were significant. The estimate of simple slope (SEs) was 0.03 (0.00) for the 400 ms ISI block, 0.05 (.00) for the 600 ms ISI block, 0.06 (.01) for the 800 ms ISI block, and 0.05 (0.01) for the 1000 ms ISI block (all *ps* < 0.001). These results indicated that the numbers of commission errors in the blocks with 600, 800, and 1000 ms ISIs in the GNG were more strongly related to CCPT performance than the number of commission errors in block with 400 ms ISI.

**Table 3:** The results of multilevel Poisson regressions examining the relationships of CCPT (Model 1) and MST performances (Model 2) with the number of commission errors in the GNG (both ns = 209)

Parameter	Estimate	*SE*	*Z*	*p*
Model 1				
Intercept	1.96	0.05	43.25	<.001
Block [400 ms ISI (0) vs. 600 ms ISI (1)]	−0.54	0.05	−10.33	<.001
Block [400 ms ISI (0) vs. 800 ms ISI (1)]	−0.97	0.06	−16.09	<.001
Block [400 ms ISI (0) vs. 1000 ms ISI (1)]	−1.21	0.06	−19.09	<.001
CCPT	0.03	0.00	7.85	<.001
Block [400 ms ISI (0) vs. 600 ms ISI (1)] × CCPT	0.02	0.00	4.81	<.001
Block [400 ms ISI (0) vs. 800 ms ISI (1)] × CCPT	0.03	0.01	5.48	<.001
Block [400 ms ISI (0) vs. 1000 ms ISI (1)] × CCPT	0.02	0.01	4.13	<.001
Model 2				
Intercept	2.02	0.05	40.76	<.001
Block [400 ms ISI (0) vs. 600 ms ISI (1)]	−0.48	0.05	−9.91	<.001
Block [400 ms ISI (0) vs. 800 ms ISI (1)]	−0.85	0.06	−14.00	<.001
Block [400 ms ISI (0) vs. 1000 ms ISI (1)]	−1.14	0.06	−17.65	<.001
MST	0.03	0.01	3.21	.001
Block [400 ms ISI (0) vs. 600 ms ISI (1)] × MST	0.02	0.01	1.91	.056
Block [400 ms ISI (0) vs. 800 ms ISI (1)] × MST	0.02	0.01	2.09	.037
Block [400 ms ISI (0) vs. 1000 ms ISI (1)] × MST	0.02	0.01	1.81	.070

Note

GNG = Go/No‐Go Task; CCPT = Conners Continuous Performance Test 3rd Edition; MST = Modified Stroop Task; ISI = Inter‐stimulus interval.

A similar multilevel Poisson regression analysis was conducted for the mean centered MST performance. As shown in Table 3, there was a significant main effect of conditions in the GNG and MST performances. Moreover, the interaction between the 400 ms ISI vs. 800 ms ISI conditions and MST performance was significant. The interaction between the 400 ms ISI vs. 600 ms ISI conditions and MST performance, and the interaction between the 400 ms ISI vs. 1000 ms ISIs conditions and MST performance indicated a significant trend. The estimate of simple slope (SEs) was 0.03 (0.01) for the 400 ms ISI block, 0.05 (0.01) for the 600 ms ISI block, 0.05 (0.01) for the 800 ms ISI block, and 0.05 (0.01) for the 1000 ms ISI block (all *ps* < 0.002). These results suggested that the number of commission errors in the 800 ISI block in the GNG was more strongly associated with MST performance than the number of commission errors in the 400 ms ISI block.

We also calculated correlation coefficients between the normally distributed number of commission errors in 400 and 600 ms ISIs blocks in the GNG and other inhibition measures. A *Z*‐test indicated that the number of commission errors in 600 ms ISI block was correlated more strongly with CCPT performance than commission errors in 400 ms ISI block (*rs* = 0.61 and 0.47, respectively, both *ps* < 0.001; *z* = 2.96, *p* = 0.004). On the other hand, correlations with MST performance were not significantly different between the numbers of commission errors in 400 and 600 ms ISIs blocks (*rs* = 0.19 and 0.26, respectively, both *ps* < .006; *z* = 1.22, *p* = 0.223). These findings were consistent with the results from the multilevel Poisson regression analyses.^2^

Finally, a one‐way Friedman test was conducted to examine the between block differences in individual mean RTs of correct go trials. This analysis showed a significant between block differences (*χ* ^2^ (3) = 51.26, *p* < 0.001). Pairwise Wilcoxon signed‐rank test with Bonferroni correction (*p* = 0.05/6 = 0.008) showed that all mean RTs differences between blocks except for the between block difference between 400 ms and 600 ms ISI were significant (*zs* > 3.06, *ps* < 0.003).^3^

## Discussion

In accordance with the findings by Young *et al*. (2018, study 1), the present study showed that the shorter the ISIs in the GNG was, the more commission errors the participants made. This finding, together with previous findings, suggest that ISI is an essential factor that determines the difficulty in the GNG. In addition, as shown in Fig. 1, the number of participants who made no commission errors decreased as ISIs shortened, thus leading to a normalized distribution of the number of commission errors. The blocks with more than 800 ms ISI seemed to be too easy for university students, leading to a highly skewed distribution of the number of commission errors. Therefore, the number of commission errors in these blocks can be considered to be undesirable for assessing individual differences. These results suggested that, in terms of the distribution of the number of commission errors, 400 ms ISI (the shortest one used in the present study) was the most desirable ISI to assess individual differences.

However, the present findings suggest that the shortest ISI has some limitations. First, the block with the 400 ms ISI produced more omission errors than the other blocks. In this block, more than half of the participants made at least one (or more) omission errors. Observation of participants experiencing the shortest ISI condition suggested that many participants were confused by the situation in which stimulus changed quickly and could not make any response in some consecutive trials. Other participants did not have sufficient time to identify whether the stimulus is a go or no‐go stimulus before it disappeared. Hence they pressed a key after the next stimulus appeared. Such observations suggested that participants might have occasionally responded (or not) without identifying what the stimulus was in each trial; if so, this prevented us from assessing response inhibition accurately by the number of commission errors in the 400 ms ISI block.

The weaker associations observed between the number of commission errors for the 400 ms ISI block in the GNG and other inhibition measures support the above interpretation. The multilevel Poisson regression analysis showed that the number of commission errors in the 400 ms ISI block in the GNG had a significantly weaker relationship with CCPT performance, which is the commonly accepted measure of response inhibition, compared to blocks with longer ISIs. It is plausible that the weaker association between the number of commission errors in the 400 ms ISI block in the GNG and CCPT performance was caused by the error variance in the number of commission errors in that block, which did not reflect response inhibition described in the previous paragraph. These findings indicate that the GNG using extremely short ISI might not be suitable for assessing response inhibition.

The results of the multilevel Poisson analyses examining between block differences in the relationship of the number of commission errors in the GNG with MST performance were in the same direction (i.e., the weakest association was found in the 400 ms ISI block in the GNG). However, the significant between block differences among the magnitudes of these associations were observed only between the 400 ms ISI and the 800 ms ISI blocks, with only a significant trend for the other between block differences. Both the GNG and the MST have traditionally been included in the list of inhibition measures (Snyder *et al*., 2015). However, recent studies have suggested that these two tasks may assess somewhat related but distinct constructs: response inhibition and attentional inhibition (Tiego *et al*., 2018).^4^ All the correlations between the number of commission errors in all the GNG blocks and MST performance were weak, which supported the model proposed by Tiego *et al*. (2018). It is plausible that these low correlations weaken the magnitudes of between block differences in the association between the number of commission errors in the four GNG blocks and MST performance.

We concluded that among 400, 600, 800, and 1000 ms ISIs in the GNG, 600 ms is the most appropriate ISI to assess individual differences of response inhibition in those settings which used white and red circles as stimuli, stimulus presentation of 250 ms, and 7:3 go/no‐go ratio. Although the block with a 600 ms ISI produced more omission errors than those with 800 and 1000 ms ISIs, the 600 ms ISI has advantages in that this distribution of commission errors is more desirable than 800 and 1000 ms ISIs. Also, the number of commission errors in the 600 ms ISI block was more strongly associated with CCPT performance than commission errors in the 400 ms ISI block. We propose that desirable ISI and other settings in the GNG should be determined by considering the number of commission errors and other information such as the number of omission errors and relationships with other inhibition measures. The number of commission errors in the appropriate ISI of the GNG is assumed to be a valid indicator of response inhibition, which could contribute to improving research on psychopathology.

The present study has specific limitations. This study examined the effects of four ISIs on each GNG variable. However, it is plausible that there are more appropriate ISIs than those used in this study. Therefore, it is suggested that future studies examine the effects of other ISIs. If there is a more suitable ISI exists, it is likely to be between 400 ms and 800 ms when other settings are held similar to those in the current study. In addition, although there are specific differences between the GNG and the CCPT including the type of stimuli, the number of go stimuli, and the fluctuation of the ISIs, these tasks had the same format consisting of go and no/go trials. It would be necessary to examine the correlation between the number of commission errors in different ISIs of the GNG and other response inhibition tasks with different formats from the GNG, such as the Stop Signal Task or the Simon Task (Tiego *et al*., 2018). Furthermore, because the participants were limited to undergraduate and graduate students aged from 18 to 26, it is unclear whether the present findings can be applied to the population with other age ranges. Replication with a population other than university students is necessary. Finally, since this study adopted a within‐subject design, a practice effect might have influenced our findings. It is desirable to assign participants to some groups that conduct the GNG with different ISIs, although between‐subject design would require researchers to recruit many more participants than was done in the present study.

## Funding

This study was supported by grants from the Japan Society for the Promotion of Science (18K13333).

## Notes

^1^ We appreciate Dr. Michael E. Young for his useful suggestions about the statistical analysis.

^2^ We also conducted a square root transformation of the numbers of commission errors in 800 and 1000 ms ISIs blocks of the GNG and calculated the correlation coefficients between these variables and two measures of inhibition. These transformed variables in 800 and 1000 ms ISIs blocks were positively correlated with CCPT performance (*rs* = 0.67 and 0.56, *p* < 0.001) and MST performances (*rs* = 0.28 and 0.29, *p* < 0.001).

^3^ The data that support the findings of this study are available on request from the corresponding author.

^4^ Other researchers have suggested that the latent variable named common executive function can be constructed from tasks assessing inhibition, updating, and shifting, and the inhibition specific factor cannot explain additional variances in tasks that are assumed to assess inhibition (Friedman & Miyake, 2017, for review). We did not discuss this further because there has been no agreement on the classification of executive functions to date.
